# Supplementary material for: Comparative genomics of Bordetella pertussis isolates from New Zealand, a country with an uncommonly high incidence of whooping cough
Source: Microb Genom. 2022 Jan 27;8(1):000756. doi: 10.1099/mgen.0.000756 (PMC8914352; doi:10.1099/mgen.0.000756)
Supplement: Supplementary material 1 [file mgen-8-0756-s001.pdf]

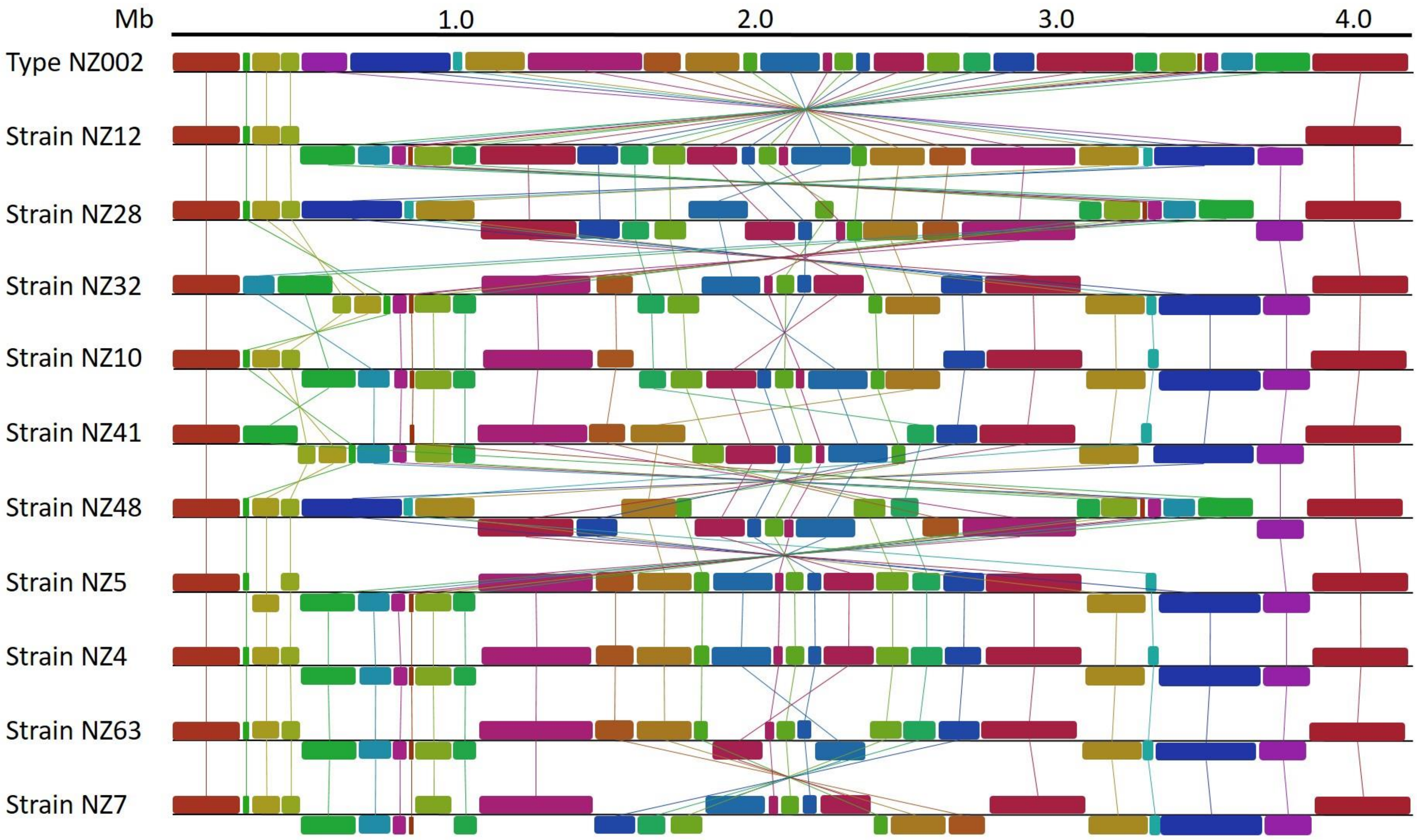

Supplementary Fig. S1. Genome structures of New Zealand "singleton" isolates

Closed genome sequences were visualised and compared using progressiveMauve. The genome structures of the ten New Zealand isolates with "singleton" arrangements, compared to the NZ002 arrangement type, are shown here. NZ48 was found to be congruent with CDC046 (not shown), whilst NZ10 was congruent with CDC Cluster-BP-23.
